# Group 3 LEA Protein, ZmLEA3, Is Involved in Protection from Low Temperature Stress

**DOI:** 10.3389/fpls.2016.01011

**Published:** 2016-07-14

**Authors:** Yang Liu, Jianan Liang, Liping Sun, Xinghong Yang, Dequan Li

**Affiliations:** ^1^State Key Laboratory of Crop Biology, Shandong Key Laboratory of Crop Biology, College of Life Sciences, Shandong Agricultural UniversityTai’an, China; ^2^Faculty of Chemistry and Chemical Engineering, Taishan Medical UniversityTai’an, China

**Keywords:** LEA protein, low-temperature stress, maize, ZmLEA3, transgenic gene

## Abstract

Late embryogenesis abundant (LEA) proteins are a family of small highly hydrophilic proteins that accumulate at the onset of seed desiccation and in response to adverse conditions such as drought, salinity, low temperature, or water deficit. In previous studies, we demonstrated that ZmLEA3 could enhance the transgenic tobacco tolerance to osmotic and oxidative stresses. Here, we demonstrated that the transcription of *ZmLEA3* in the maize stems could be significantly induced by low temperature and osmotic stresses and by treatment with abscisic acid (ABA) and H_2_O_2_. Further study indicated that ZmLEA3 is a single copy gene in the maize genome. The ZmLEA3 protein could protect lactate dehydrogenase (LDH) activity at low temperatures. The overexpression of *ZmLEA3* conferred tolerance to low-temperature stress to transgenic tobacco, yeast (GS115) and *E. coli* (BL21).

## Introduction

Low-temperature damage is the main factor limiting plant growth and crop production. Plants have developed a wide variety of mechanisms to cope with environmental stresses. Accumulation of the late embryogenesis abundant (LEA) proteins is an important response to adverse conditions. LEA proteins accumulate in abundance during the late development stage of seeds (Dure et al., 1989; Tunnacliffe and Wise, 2007; Kosová et al., 2014; Chiappetta et al., 2015). Although the mechanistic basis for such protection is unclear, several studies have demonstrated that LEA proteins may protect cells during desiccation and freezing by acting as hydration buffers, sequestering ions and stabilizing proteins, membranes and chromatin structures (Amara et al., 2013; Battaglia and Covarrubias, 2013; Yang et al., 2015).

The LEA proteins can be classified into seven groups, in which the group 3 LEA proteins are characterized by a repeating motif of 11 amino acids (TAQAAKEKAGE; Dure, 1993). Because the number of repetitions of the 11-mer motif is different, the molecular mass in this group of proteins varies (Battaglia et al., 2008). Bioinformatics analysis indicated that the 11-mer exists principally as an amphipathic α-helix. In previous studies, we have demonstrated that ZmLEA3 could enhance transgenic tobacco tolerance to osmotic and oxidative stresses. Although the functions of the group 3 LEA proteins remain unknown, they are assumed to be important for the establishment of environmental stress tolerance in seeds. Overexpressing wheat TaLEA3 and TaLEA2 in yeast improved transgenic yeast tolerance to hyperosmotic, salt, and freezing stresses (Yu et al., 2005). The synthetic peptide PvLEA-22 has the ability to preserve liposomes in the dry state (Furuki and Sakurai, 2014).

Soybean group 3 LEA protein PM2 (seed maturation protein) conferred the tolerance to the *E. coli* recombinant against diverse stresses by protecting proteins and enzyme activity under low- or high-temperature conditions, and the 22-mer repeat region is an important functional region (Liu and Zheng, 2005; Liu et al., 2010).

LEA proteins were first found in cotton seeds and accumulated to high levels during the last stage of seed maturation and in water deficits in vegetative plant tissues (Dure et al., 1981). Many studies have reported that LEA proteins can be involved in abiotic and biotic stresses. Although some mechanisms have been proposed, the functional mechanism remains unclear (Brini et al., 2011; Eriksson et al., 2011; Petersen et al., 2012; Salleh et al., 2012). Previously, we demonstrated that the overexpression of *ZmLEA3* in tobacco resulted in improved osmotic stress tolerance and enhanced tolerance for oxidation stress (Liu et al., 2013). In the present study, we showed that the ZmLEA3 protein could protect enzymes from damage caused by low temperature. Further study indicated that the overexpression of ZmLEA3 conferred low-temperature stress tolerance in transgenic lines.

## Materials and Methods

### Plant Materials and Growth Condition

The tobacco plants (*Nicotiana tabacum* cv NC 89) was used in this study. The tobacco seeds were plated on Murashige and Skoog (MS) medium (Murashige and Skoog, 1962) containing 200 mg L^-1^ kanamycin under light/dark cycle conditions of 16/8 h at 25°C. For the low-temperature treatment, 6-week-old transgenic tobacco lines were treated at 4°C under the same light periods. Samples were collected at the indicated time after the treatments, and all samples were frozen in liquid nitrogen immediately after collection and stored at -80°C.

### Bioinformatic Analysis

Sequence identities were determined using BLAST on the NCBI web server^1^ and MaizeGDB^2^. The subcellular localizations were determined by means of the ProtComp v. 9.0^3^. The isoelectric point (p*I*), molecular weight (MW), and grand average hydropathy (GRAVY) values were estimated by the ProtParam tool^4^.

### Expression Profiles of *ZmLEA3* in Stem under Different Stress Treatments Using Real-Time PCR

Two-week-old maize was treated with 100 μM abscisic acid (ABA), 20% polyethylene glycol (PEG) 6000 (w/v), 20 μM H_2_O_2_, and low temperature (4°C). Stem tissues collected from the various treated plants at specific time points were immediately frozen in liquid nitrogen and stored at -80°C. The plants treated with only water were used as the experimental controls.

### Southern Blot Hybridizations

Ten micrograms of genomic DNA from maize was digested by *Eco*RI, *Eco*RV, or *Bam*HI restriction endonucleases. The restricted genomic DNA was separated on a 1.0% agarose gel and transferred to nylon membranes (Hybond-N^+^, Amersham) by capillary method using 20× saline sodium citrate (SSC). The restricted DNA was subsequently immobilized on the membrane by baking at 120°C for 30 min. The coding sequence of the ZmLEA3 gene was used as a probe, which was labeled with [α-^32^P] dATP using a random-primed method.

### *In Vitro* Stress Assays and LDH Activity Measurements

Lactate dehydrogenase (LDH) (Roche, UK) from rabbit muscle was diluted in 25 mM Tris–HCl, pH 7.5. The low-temperature treatment was assayed according to the previously published method (Reyes et al., 2008). The mixture in a final volume of 75 μl was frozen for 15 min in dry ice and thawed for 15 min in a water bath at 25°C. The final enzyme concentration was 200 nM. For LDH activity, the assay buffer was 25 mM Tris–HCl pH 7.5 containing 2 mM pyruvate (Sigma) and 0.15 mM NADH (dihydronicotinamide-adenine dinucleotide, Roche). LDH activity was monitored as the rate of decrease in absorbance at 340 nm for 1 min due to the conversion of NADH into NAD at 25°C. The rate determined for the untreated samples was considered to be 100% in all graphs.

### Expression of ZmLEA3 in Pichia Yeast GS115 and *E. coli* BL21

The transgenic yeast (Ppic3.5K-ZmLEA3) and *E. coli* (PET30-ZmLEA3) were kept in our labs. The *ZmLEA3* gene was inserted into the pET30 vector (pET system) and then transformed into *E. coli* BL21 Star cells to create the BL/ZmLEA3 recombinant. The expression of recombinant proteins with 6× His tag at N-terminus was performed following the manufacturer’s protocol (pET system). The Pichia GS115 strain was transformed with 5 μg of linearized plasmid by the LiCl method according to the EasySelect Pichia Expression Kit (Invitrogen, USA).

### Low-Temperature Tolerance Assays of Yeast and *E. coli* Transformants

Low-temperature tolerance assays were performed as described previously (Liu et al., 2014). For yeast, the recombinant yeast was inoculated in 25 ml BMGY (buffered glycerol-complex medium, 1% yeast extract, 2% peptone, 1.34% yeast nitrogen base (YNB), 10 mM K_3_PO_4_, 4 × 10^-5^ mM biotin, and 1% glycerin). After 18 h of incubation at 28°C, the cells were collected by centrifugation and resuspended in 200 ml induction BMMY (buffered methanol-complex medium, 1% yeast extract, 2% peptone, 10 mM K_3_PO_4_, 1.34% YNB, 4 × 10^-5^ mM biotin, and 0.5% methanol) and then incubated at 28°C for 4 days. Methanol was added every 24 h to a final concentration of 0.5%. To assay the low-temperature tolerance of the *E. coli* recombinant, the cultures were induced with isopropyl β-D-1-thiogalactopyranoside (IPTG, 1 mM) for 4 h.

For low-temperature treatment, the culture (yeast or *E. coli*, 1 ml) was cooled at -20°C for 24 h and then inoculated into 150 ml of BMGY or lysogeny broth (LB) medium, respectively. At each time point, 3 ml of culture was used to measure the OD_600_ with a spectrophotometer.

### Overexpression of *ZmLEA3* in Tobacco

The coding regions of *ZmLEA3* were ligated into the binary pBI121 expression vector under the CaMV 35S (promoter of cauliflower mosaic virus) promoter. Constructs were transformed into *Agrobacterium tumefaciens* strain LBA4404 and then transformed into tobacco plants by using the leaf disc transformation method.

### Germination Experiments

Approximately 50 surface-sterilized seeds from each homozygous T2 transgenic line and the wild-type (WT) tobacco line were plated on solid media composed of MS basal salts and 0.8% sucrose. To determine cold-stress sensitivity, the plates were incubated in a controlled-environment growth chamber at 12°C with a photoperiod of 16/8 h (day/night). Thirty days later, the rates of seed germination were evaluated (root emergence). Each experiment was repeated a minimum of three times with identical results.

### Low-Temperature Stress Treatments and Assays in Plants

Six-week-old tobacco seedlings were kept at 4°C for the indicated time, and then 0.5 g of leaves was collected for malondialdehyde (MDA) and relative electrolytic leakage measurements, which were performed as described previously (Liu et al., 2013; Wang et al., 2014). The activity of superoxide dismutase (SOD) and peroxidase (POD) measurements that were performed as described previously (Jiang and Zhang, 2001).

### Statistical Analysis

Statistical analyses and plotting were performed using SigmaPlot and SPSS software. The statistical significance of the difference was confirmed by Student’s *t*-test, ^∗^*P* < 0.05; ^∗∗^*P* < 0.01.

## Results and Discussion

### The Group 3 LEA Proteins Encoding Genes in the Maize Genome

The LEA protein family is very large in plants, with up to 51 genes encoding LEA proteins in *Arabidopsis thaliana* (Hundertmark and Hincha, 2008). Using the group 3 LEA protein genes from *A. thaliana*, we performed BLAST searches against the maize genomic database. According to the results, this analysis identified seven putative group 3 LEA protein genes in the complete maize genome (**Table 1**). All of these proteins were highly hydrophobic with grand average hydrophobicities from -1.024 to -0.796 and theoretical p*I* values from 6.16 to 8.98. The MWs of these proteins ranged from 18.6 to 61.8 kDa. We also predicted the subcellular localizations by means of ProtComp v.9.0. The results indicated that the group 3 LEA proteins were localized in the nucleus, cytoplasm, plasma membrane, and chloroplast membrane.

**Table 1:** Characteristics of the group 3 LEA genes identified in maize.

Name	Accession no.	Chromosome localization	Length	MW	GRAVY	p*I*	Intron	Location
ZmLEA3-1	NM_001153473.1	6:161966271-161967560	182	18599	-1.102	7.84	2	Mitochondrial or nuclear
ZmLEA3-2	BT062068.2	10:117570524-117572326	354	37909	-1.036	6.6	1	Cytoplasmic
ZmLEA3-3	NM_001111828.1	9:133421515-133422685	221	22759	-1.204	8.8	1	Cytoplasmic and nuclear
ZmLEA3-4	NM_001156540.1	6:161966444-161966664	320	33572	-0.932	6.16	0	Plasma membrane
ZmLEA3-5	NM_001156540.1	1:14450012-14450363	305	32278	-0.887	8.39	0	Nuclear
ZmLEA3-6	EU952743.1	2:10718061-10178389	205	21190	-0.796	8.98	0	Cytoplasmic
ZmLEA3-7	AFW71082.1	5:142403718-142404354	604	61763	-0.814	7.29	0	Chloroplast membrane

### Accumulation of the *ZmLEA3* Transcript under Different Stress Treatments

**FIGURE 1:**
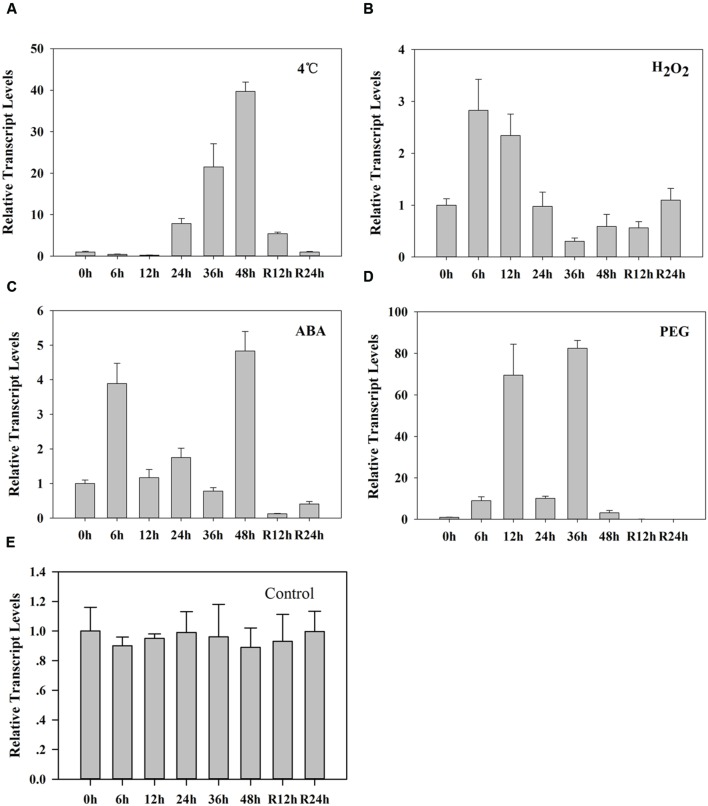
**Transcript accumulation of *ZmLEA3* in maize stems**. The maize seedlings were incubated with Hoagland’s solution for 2 weeks; uniformly sized plants at similar growth stages were chosen for further study. The total RNA was isolated from the stems. Maize seedlings were treated with 4°C **(A)**, 10 mM H_2_O_2_ **(B)**, 100 μM abscisic acid (ABA) **(C)**, 20% polyethylene glycol (PEG) 6000 (w/v) **(D)**, or water (control) **(E)**. Total RNA was isolated from the stems at the indicated times after the treatments. Each curve/column represents an average of three replicates, and error bars represent standard deviation. R represents removal of the treatment.

A previous study has demonstrated that the transcription of *ZmLEA3* was higher in the stems than in the roots and leaves (Liu et al., 2013). To further determine the expression pattern of *ZmLEA3*, quantitative real-time reverse transcription-PCR (qRT-PCR) was performed to examine the transcript levels of *ZmLEA3* in the maize stems. The results demonstrated that the transcript accumulation of *ZmLEA3* could be induced by dehydration and low temperature as well as by treatment with ABA and H_2_O_2_ (**Figure 1**). In the low-temperature treatment, the transcript level of *ZmLEA3* reached its highest level suddenly at 48 h and then reverted back to near its control level immediately when the signal was removed. The transcript accumulation of *ZmLEA3* in response to H_2_O_2_ reached its highest level at 6 h and then was reduced to its usual normal level. The transcript level of *ZmLEA3* in response to ABA treatment reached a high peak within 6 h and then was reduced at 12 h, but its transcript accumulation reached its highest level at 48 h and decreased to close to its uninduced level when the treatment was removed. For 20% PEG treatment, there were two peaks, with the transcript accumulation reaching a high level at 12 and 36 h, suggesting the existence of a feedback adjustment.

### *ZmLEA3* is a Single Copy Gene in the *Zea mays* Genome

**FIGURE 2:**
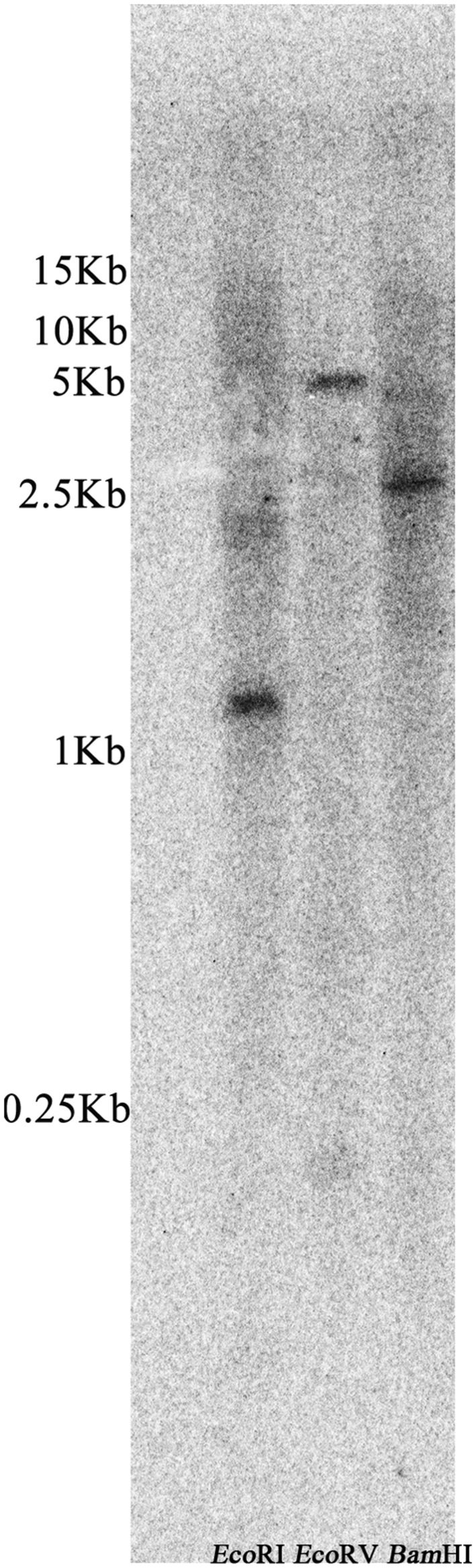
**Southern blot analysis of the *ZmLEA3* gene from maize**. Genomic DNA isolated from young leaves was restricted with *Eco*RI, *Eco*RV, and *Bam*HI before being filter hybridized with [α-^32^P] dATP-labeled probes against *ZmLEA3* sequence.

Southern blot analysis was performed to investigate whether the homology gene of *ZmLEA3* was present in the *Z. mays* genome. As shown in **Figure 2**, only one hybridization band of the digested fragments was observed with the probe. This result indicated that ZmLEA3 is a single copy gene in the *Z. mays* genome.

### ZmLEA3 Protein Protected LDH Activity from Damage Caused by Low-Temperature Stress

**FIGURE 3:**
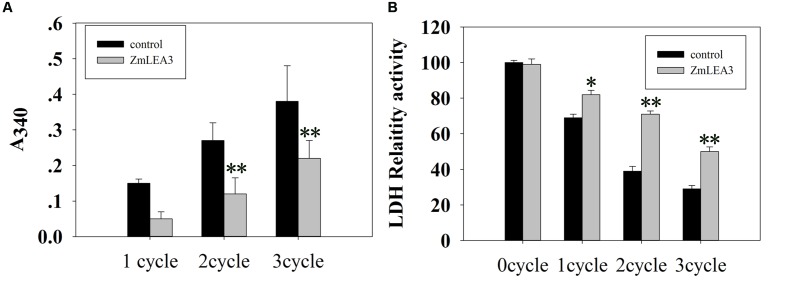
**The ZmLEA3 protein could protect lactate dehydrogenase (LDH) from damage caused by low-temperature stress**. LDH (Roche, UK) from rabbit muscle was diluted in 25 mM Tris–HCl, pH 7.5. **(A)** Aggregation of LDH after low-temperature stress in the presence of 0.24 mg of ZmLEA3 or bovine serum albumin (BSA). Aggregation was measured by the effect of light scattering giving an apparent A_340_ in the spectrophotometer. **(B)** The effect of low temperature on LDH activity. Each curve/column represents an average of three replicates, and error bars represent standard deviation. The data of the control plant and the transgenic plant were compared for the significance difference at each respective time point, ^∗^*P* < 0.05, ^∗∗^*P* < 0.01.

It has been previously demonstrated that freeze–thaw can reduce LDH activity (Reyes et al., 2008). To determine the anti-aggregation activity of the ZmLEA3 protein, an assay was used to measure the protective effect of ZmLEA3 proteins on LDH activity. As shown in **Figure 3**, ZmLEA3 could protect the activity and reduce the aggregation of LDH under freeze–thaw cycle.

LDH as a reporter enzyme had been used to test the protection activity of different LEA proteins from different groups and species (Tunnacliffe and Wise, 2007). It has been hypothesized that LEA proteins may play protective roles for proteins by acting as chaperone molecules (Kovacs et al., 2008). In this report, we demonstrated that ZmLEA3 could protect the LDH activity from damage caused by freeze–thaw cycle. Hernández-Sánchez et al. (2014) reported that LEA proteins could function through the formation of dimers and large multimeric complexes, forming a large molecular shield around its biological targets. These results indicated that LEA proteins could prevent protein aggregation under environmental stresses by stabilizing protein species in a partially unfolded state. According to these results, we demonstrated that ZmLEA3 proteins could protect the activity of protein from damage caused by low temperature.

### Overexpression ZmLEA3 Conferred Tolerance to Low Temperature on Transgenic Yeast and *E. coli*

**FIGURE 4:**
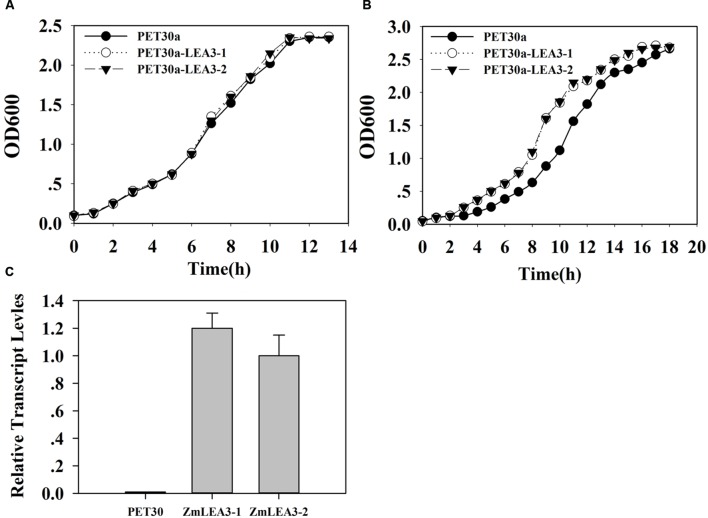
**Overexpression enhances tolerance to low-temperature stress in the *E. coli ZmLEA3* transformant**. **(A)** The transformant *E. coli* (BL21) with the empty vector or PET30-*ZmLEA3* were grown in lysogeny broth (LB) medium. **(B)** The transformant *E. coli* (BL21) were grown in the non-stress LB medium after -20°C treatment. Two yeast and *E. coli* transformants were chosen for further study. **(C)** Accumulation of the *ZmLEA3* in transgenic *E. coli*. Each curve/column represents an average of three replicates, and error bars represent standard deviation.

**FIGURE 5:**
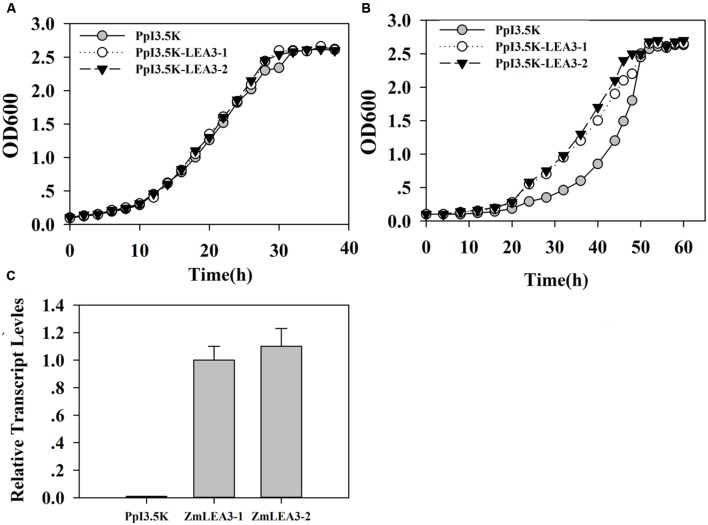
**Overexpression enhances tolerance to low-temperature stress in the yeast *ZmLEA3* transformant**. **(A)** The transformant yeasts (GS115) with the empty vector or PpI3.5K-*ZmLEA3* were grown in the non-stress buffered glycerol-complex medium (BMGY). **(B)** The transformant yeasts were grown in the non-stress BMGY medium after -20°C treatment. **(C)** Accumulation of the *ZmLEA3* in transgenic yeasts. Each curve/column represents an average of three replicates, and error bars represent standard deviation.

To determine the function of the ZmLEA3 fusion protein in low-temperature stress, the effects of low temperature on the growth of the recombinant *E. coli* and yeast were examined under low-temperature stress. The growth curves of the yeast and *E. coli* cell lines transformed with the pPI3.5k-*ZmLEA5C* or PET30-ZmLEA3 vector and the control lines containing the empty vector (pPI3.5k or PET30) were measured under low-temperature stress. Under optimal conditions, there was no significant difference between the transformed lines and the control. However, under low-temperature stress, the transformed yeast and *E. coli* displayed higher growth compared with the control, and the lag phase of the transformed yeasts and *E. coli* was shorter than that the control (**Figures 4** and **5**).

The phosphorylation and dephosphorylation of LEA protein seems to be a major post-translational modification (Rorat, 2006). The phosphorylation of maize dehydrin Rab17 by protein kinase CKII is the relevant step for its nuclear localization (Jensen et al., 1998). Jensen et al. (1998) reported that phosphorylation regulated ion-binding is a property shared by the acidic subclass dehydrins. Because recombinant protein is not phosphorylated by *E. coli*, the phosphorylation did not influence the function of ZmLEA3. Here, our results indicated that overexpression of ZmLEA3 could enhance the tolerance to low-temperature in transgenic yeast and *E. coli*. This result is consistent with a previous study, in which a group 3 LEA protein was not phosphorylated by protein kinase (Amara et al., 2012).

### Overexpression of ZmLEA3 Enhanced Transgenic Tobacco Tolerance to Low-Temperature Stress

**FIGURE 6:**
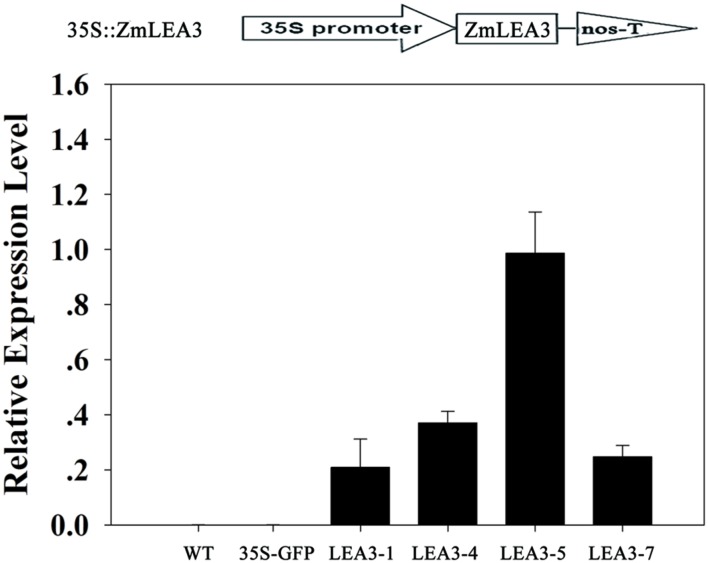
***ZmLEA3* transcript accumulation in transgenic tobacco plants**. Expression of *ZmLEA3* in tobacco plants was analyzed by qRT-PCR. Total RNA was isolated from leaf samples collected from T2 tobacco plants.

**FIGURE 7:**
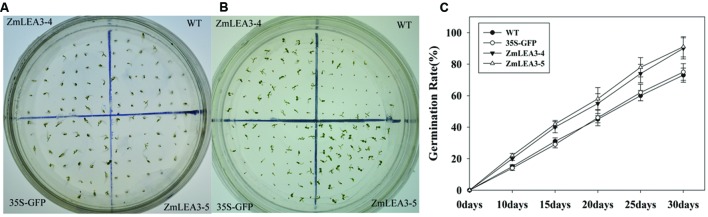
**The primary analysis of the low-temperature stress tolerance in transgenic tobacco plants**. The phenotype of transgenic and control tobacco seeds grown for 4 weeks under normal condition **(A)** or low-temperature (**B**, 12°C) stress. **(C)** The seed germination rate of the tobacco seeds under low-temperature (12°C) stress.

A previous study indicated that ZmLEA3 could enhance tolerance to drought and oxidative stresses in transgenic tobacco and yeast (Liu et al., 2013). To further address the function of the ZmLEA3 protein, the low-temperature tolerance of the transgenic tobacco was analyzed. Independent transgenic lines were obtained by kanamycin-resistance selection and confirmed by genomic PCR (data not shown). The expression levels of *ZmLEA3* in tobacco were detected by qRT-PCR, whereas no expression was detected in the control plants (WT and 35S-GFP/green fluorescent protein). Two (LEA3-4 and LEA3-5) of the *ZmLEA3* transgenic lines were chosen for further analysis (**Figure 6**). As shown in **Figure 7A**, there were no differences in the germination rate between the transgenic lines and the control plants (WT and 35S-GFP) under the control conditions. However, the germination rate of the transgenic tobacco plants was higher than that of the control plants under low-temperature stress (**Figures 7B,C**).

**FIGURE 8:**
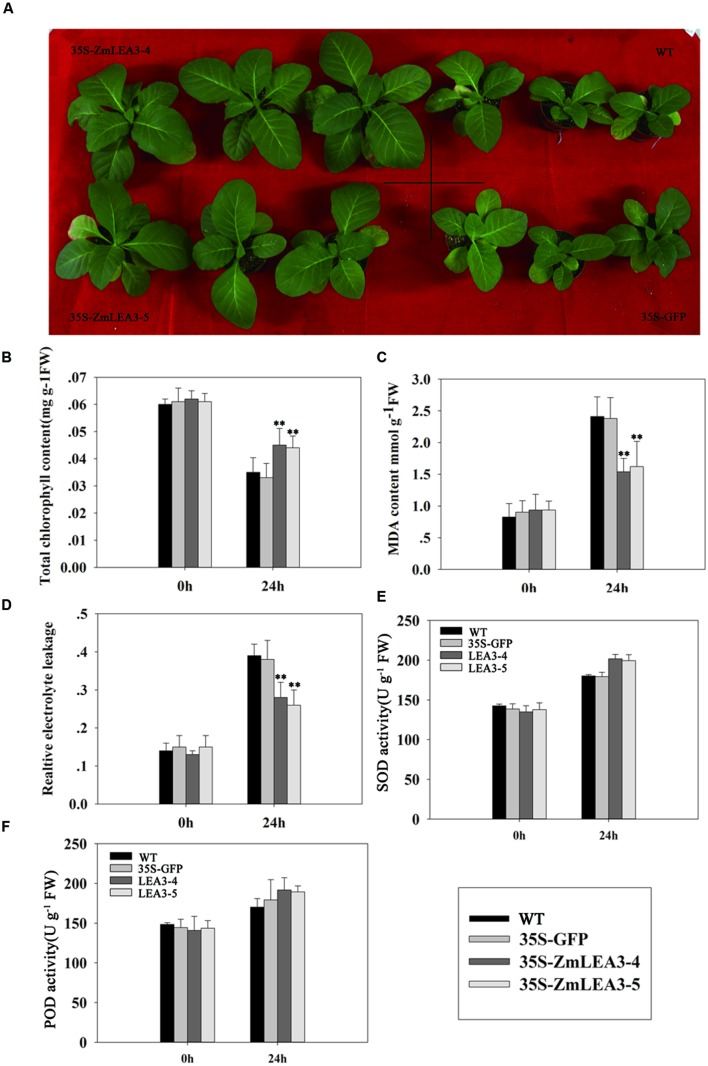
**The low-temperature tolerance of transgenic and control tobacco plants**. The phenotype of transgenic and control tobacco plants grown in normal conditions after low-temperature treatment **(A)**. Total chlorophyll content **(B)**, malondialdehyde (MDA) **(C)**, the relative electrolyte leakage **(D)**, the activity of superoxide dismutase (SOD) **(E),** and peroxidase (POD) **(F)** were measured in 6-week-old transgenic and control tobacco plants before and after low-temperature treatment (4°C) at the indicated time. Each curve/column represents an average of three replicates, and error bars represent standard deviation. The statistical significance of the difference was confirmed by Student’s *t*-test, ^∗∗^*P* < 0.01.

Several reports have demonstrated that LEA proteins enhance transgenic plants low-temperature tolerance, but there is little known about the roles of the maize group 3 LEA proteins under low-temperature stress. Yu et al. (2005) reported that overexpression of the group 3 LEA proteins *TaLEA2* and *TaLEA3* improved transgenic yeast cell tolerance to low-temperature stress, and yeast transformants with *TaLEA2* seemed to be more tolerant to freezing stress than transformants with *TaLEA3*. Overexpression of the barley HVA1 proteins in *Saccharomyces cerevisiae* resulted in the generation of freezing-tolerant organisms (Zhang et al., 2000). To investigate the low-temperature tolerance of the transgenic plants, the tobacco plants were treated at 4°C for 24 h and were then transferred to normal conditions. As shown in **Figure 8A** that the transgenic plants grew significantly better than the control plants. Chlorophyll content can be used to estimate the degree of leaf senescence. Under normal conditions, there is no significant difference in the chlorophyll levels, but the chlorophyll content was also significantly reduced in the control plants compared with the transgenic plants (**Figure 8B**). MDA content and the relative electrolyte leakage can represent the membrane injury. In this study, our results indicated that cold stress significantly increased MDA content and relative electrolyte leakage in the control plants compared with the transgenic plants (**Figures 8C,D**).

Antioxidant enzymes scavenge reactive oxygen species (ROS), which can accumulate when stress is imposed. Based on the above results that the ZmLEA3 protein could protect the activity of LDH in low-temperature stress, we speculated that ZmLEA3 could protect antioxidant enzymes such as SOD or POD. To confirm this hypothesis, we checked the activity of SOD and POD. As shown in **Figures 8E,F**, the activity of SOD and POD was higher in the transgenic lines than in the control plants after low-temperature treatment.

## Conclusion

In summary, these findings provide evidence that ZmLEA3 is a single copy gene in the *Z. mays* genome and plays an important role in conferring low-temperature tolerance in prokaryotes and eukaryotes. We propose that the group 3 LEA protein may serve as a membrane and protein stabilizer during low-temperature stress. However, the protective role has not been completely elucidated, and further experiments are necessary to explore the molecular mechanism.

## Author Contributions

YL and DL designed the experiments. YL performed the experiments and analyzed the data. YL and JL wrote the article. LS and XY gave positive suggestion about this article. All authors read and approved the manuscript.

## Conflict of Interest Statement

The authors declare that the research was conducted in the absence of any commercial or financial relationships that could be construed as a potential conflict of interest.

## Footnotes

1 http://blast.ncbi.nlm.nih.gov/Blast.cgi

2 http://www.maizegdb.org/popcorn/search/sequence_search/home.php?a=BLAST_UI

3 http://linux1.softberry.com/berry.phtml?topic=protcomppl\&group=Programs\&subgroup=proloc

4 http://us.expasy.org/tools/protparam.html

## References

[B1] Amara I. Capellades M. Ludevid M. D. Pagès M. Goday A. (2013). Enhanced water stress tolerance of transgenic maize plants over-expressing LEA Rab28 gene. *J. Plant Physiol.* 170 864–873. 10.1016/j.jplph.2013.01.004 23384757

[B2] Amara I. Odena A. Oliveira E. Moreno A. Masmoudi K. Pagés M. (2012). Insights into maize LEA proteins: from proteomics to functional approaches. *Plant Cell Physiol.* 53 312–329. 10.1093/pcp/pcr183 22199372

[B3] Battaglia M. Covarrubias A. A. (2013). Late embryogenesis abundant (LEA) proteins in legumes. *Front. Plant Sci.* 4:190 10.3389/fpls.2013.00190 PMC3691520 23805145

[B4] Battaglia M. Olvera-Carrillo Y. Garciarrubio A. Campos F. Covarrubias A. A. (2008). The enigmatic LEA proteins and other hydrophilins. *Plant Physiol.* 148 6–24. 10.1104/pp.108.120725 18772351 PMC2528095

[B5] Brini F. Yamamoto A. Jlaiel L. Takeda S. Hobo T. Dinh H. Q. (2011). Pleiotropic effects of the wheat dehydrin DHN-5 on stress responses in *Arabidopsis*. *Plant Cell Physiol.* 52 676–688. 10.1093/pcp/pcr030 21421569

[B6] Chiappetta A. Muto A. Bruno L. Woloszynska M. Van Lijsebettens M. Bitonti M. B. (2015). A dehydrin gene isolated from feral olive enhances drought tolerance in *Arabidopsis* transgenic plants. *Front. Plant Sci.* 6:392 10.3389/fpls.2015.00392 PMC4485055 26175736

[B7] Dure L. (1993). A repeating 11-mer amino acid motif and plant desiccation. *Plant J.* 3 363–369. 10.1046/j.1365-313X.1993.t01-19-00999.x 8220448

[B8] Dure L. Crouch M. Harada J. Ho T. H. D. Mundy J. Quatrano R. (1989). Common amino acid sequence domains among the LEA proteins of higher plants. *Plant Mol. Biol.* 12 475–486. 10.1007/BF00036962 24271064

[B9] Dure L. Greenway S. C. Galau G. A. (1981). Developmental biochemistry of cottonseed embryogenesis and germination: changing messenger ribonucleic acid populations as shown by in vitro and in vivo protein synthesis. *Biochemistry* 20 4162–4168. 10.1021/bi00517a033 7284317

[B10] Eriksson S. K. Kutzer M. Procek J. Grobner G. Harryson P. (2011). Tunable membrane binding of the intrinsically disordered dehydrin Lti30, a cold-induced plant stress protein. *Plant Cell* 23 2391–2404. 10.1105/tpc.111.085183 21665998 PMC3160030

[B11] Furuki T. Sakurai M. (2014). Group 3 LEA protein model peptides protect liposomes during desiccation. *Biochim. Biophys. Acta* 1838 2757–2766. 10.1016/j.bbamem.2014.07.009 25037007

[B12] Hernández-Sánchez I. E. Martynowicz D. M. Rodríguez-Hernández A. A. Pérez-Morales M. B. Graether S. P. Jiménez-Bremont J. F. (2014). A dehydrin-dehydrin interaction: the case of SK3 from *Opuntia streptacantha*. *Front. Plant Sci.* 5:520 10.3389/fpls.2014.00520 PMC4193212 25346739

[B13] Hundertmark M. Hincha D. K. (2008). LEA (late embryogenesis abundant) proteins and their encoding genes in *Arabidopsis thaliana*. *BMC Genomics* 9:118 10.1186/1471-2164-9-118 PMC2292704 18318901

[B14] Jensen A. B. Goday A. Figueras M. Jessop A. C. (1998). Phosphorylation mediates the nuclear targeting of the maize Rab17 protein. *Plant J.* 13 691–697. 10.1046/j.1365-313X.1998.00069.x 9681011

[B15] Jiang M. Zhang J. (2001). Effect of abscisic acid on active oxygen species, antioxidative defence system and oxidative damage in leaves of maize seedlings. *Plant Cell Physiol.* 42 1265–1273. 10.1093/pcp/pce162 11726712

[B16] Kosová K. Vítámvás P. Prášil I. T. (2014). Wheat and barley dehydrins under cold, drought, and salinity–what can LEA-II proteins tell us about plant stress response? *Front. Plant Sci*. 5:343 10.3389/fpls.2014.00343 PMC4089117 25071816

[B17] Kovacs D. Kalmar E. Torok Z. Tompa P. (2008). Chaperone activity of ERD10 and ERD14, two disordered stress-related plant proteins. *Plant Physiol.* 147 381–390. 10.1104/pp.108.118208 18359842 PMC2330285

[B18] Liu Y. Wang L. Jiang S. S. Pan J. W. Cai G. H. Li D. Q. (2014). Group 5 LEA protein, ZmLEA5C, enhance tolerance to osmotic and low temperature stresses in transgenic tobacco and yeast. *Plant Physiol. Biochem.* 84 22–31. 10.1016/j.plaphy.2014.08.016 25240107

[B19] Liu Y. Wang L. Xing X. Sun L. Pan J. Kong X. (2013). ZmLEA3, a multifunctional group 3 LEA protein from maize (*Zea mays* L.), is involved in biotic and abiotic stresses. *Plant Cell Physiol.* 54 944–959. 10.1093/pcp/pct047 23543751

[B20] Liu Y. Zheng Y. (2005). PM2, a group 3 LEA protein from soybean, and its 22-mer repeating region confer salt tolerance in *Escherichia coli*. *Biochem. Biophys. Res. Commun.* 331 325–332. 10.1016/j.bbrc.2005.03.165 15929202

[B21] Liu Y. Zheng Y. Zhang Y. Wang W. Li R. (2010). Soybean PM2 protein (LEA3) confers the tolerance of *Escherichia coli* and stabilization of enzyme activity under diverse stresses. *Curr. Microbiol.* 60 373–378. 10.1007/s00284-009-9552-2 19949793

[B22] Murashige T. Skoog F. (1962). A revised medium for rapid growth and bioassay with tobacco tissue cultures. *Physiol. Plant* 15 473–497. 10.1111/j.1399-3054.1962.tb08052.x

[B23] Petersen J. Eriksson S. K. Harryson P. Pierog S. Colby T. Bartels D. (2012). The lysine-rich motif of intrinsically disordered stress protein CDeT11-24 from *Craterostigma plantagineum* is responsible for phosphatidic acid binding and protection of enzymes from damaging effects caused by desiccation. *J. Exp. Bot.* 63 4919–4929. 10.1093/jxb/ers173 22791833 PMC3428009

[B24] Reyes J. L. Campos F. Wei H. Arora R. Yang Y. Karlson D. (2008). Functional dissection of hydrophilins during in vitro freeze protection. *Plant Cell Environ.* 31 1781–1790. 10.1111/j.1365-3040.2008.01879.x 18761701

[B25] Rorat T. (2006). Plant dehydrins—tissue location, structure and function. *Cell Mol. Biol. Lett.* 11 536–556. 10.2478/s11658-006-0044-0 16983453 PMC6275985

[B26] Salleh F. M. Evans K. Goodall B. Machin H. Mowla S. B. Mur L. A. J. (2012). A novel function for a redox-related LEA protein (SAG21/AtLEA5) in root development and biotic stress responses. *Plant Cell Environ.* 35 418–429. 10.1111/j.1365-3040.2011.02394.x 21736589

[B27] Tunnacliffe A. Wise M. J. (2007). The continuing conundrum of LEA proteins. *Naturwissenschaften* 94 791–812. 10.1007/s00114-007-0254-y 17479232

[B28] Wang L. Liu Y. Cai G. H. Jiang S. S. Pan J. W. Li D. Q. (2014). Ectopic expression of ZmSIMK1 leads to improved drought tolerance and activation of systematic acquired resistance in transgenic tobacco. *J. Biotechnol.* 172 18–29. 10.1016/j.jbiotec.2013.11.006 24291188

[B29] Yang W. Zhang L. Lv H. Li H. Zhang Y. Xu Y. (2015). The K-segments of wheat dehydrin WZY2 are essential for its protective functions under temperature stress. *Front. Plant Sci.* 6:406 10.3389/fpls.2015.00406 PMC4467595 26124763

[B30] Yu J. N. Zhang J. S. Shan L. Chen S. Y. (2005). Two new group 3 LEA genes of wheat and their functional analysis in yeast. *J. Integr. Plant Biol.* 47 1372–1381. 10.1111/j.1744-7909.2005.00126.x

[B31] Zhang L. Ohta A. Takagi M. Imai R. (2000). Expression of plant group 2 and group 3 lea genes in *Saccharomyces cerevisiae* revealed functional divergence among LEA proteins. *J. Biochem.* 127 611–616. 10.1093/oxfordjournals.jbchem.a022648 10739953

